# Investigation on the impact of quality characteristics and storage stability of foxtail millet induced by air cold plasma

**DOI:** 10.3389/fnut.2022.1064812

**Published:** 2022-12-07

**Authors:** Lang-Hong Wang, Zhongyan Li, Jiale Qin, Yanyan Huang, Xin-An Zeng, Rana Muhammad Aadil

**Affiliations:** ^1^Guangdong Provincial Key Laboratory of Intelligent Food Manufacturing, School of Food Science and Engineering, Foshan University, Foshan, China; ^2^College of Food Science and Technology, Northwest University, Xi’an, China; ^3^School of Food Science and Engineering, South China University of Technology, Guangzhou, China; ^4^National Institute of Food Science and Technology, University of Agriculture, Faisalabad, Pakistan

**Keywords:** foxtail millet, dielectric barrier discharge-air cold plasma, lipoxygenase, lipase, accelerated storage

## Abstract

The aim of this work was to investigate the effects of dielectric barrier discharge-air cold plasma (DBD-ACP, 15–35 kV, 2–12 min) on the quality of foxtail millets. The *L* and b* values were evaluated by a digital colorimeter representing that the color of millets was significantly changed at 25 kV for 4–12 min or at 35 kV for 2–12 min. The results were consistent with the change of total yellow pigment in millets, indicating that DBD-ACP damaged the carotenoids if the treatment condition was too high. The activity of lipoxygenase and lipase, involving the oxidation and hydrolysis of lipids of millet, decreased significantly induced by DBD-ACP. For example, the lipoxygenase and lipase activity of Mizhi millet was decreased from 44.0 to 18.7 U g^–1^min^–1^, 56.0–15.1 U/(mg pro) (*p*<0.05) after being exposed to 25 kV for 2–12 min, respectively. Changes of color, lipoxygenase and lipase activity, and malondialdehyde content of millets were determined during accelerated storage (40 ± 2°C and 75% Relative Humidity) for 15 days after being treated by DBD-ACP under 15 and 25 kV for 4 min. Results showed that millets treated by DBD-ACP at 15 kV kept a better color with lower malondialdehyde content, and lower lipoxygenase and lipase activity compared to control. This work implied that DBD-ACP is an underlying approach for the storage of foxtail millets.

## Introduction

Foxtail millet (*Setaria italica*) has a long planting history in China as one of the essential coarse grain crops, especially in the northern area (1). Currently, the cultivation area of foxtail millet reaches 10.57 × 10^6^ ha worldwide, with a high production of about 2.29 × 10^6^ t (2). This kind of grain crop is rich in nutritional components, comprising 12.5% protein, 8.0% crude fiber, 4.3% fat content, 3.3% ash content, and around 61% carbohydrates (3). It is deemed a medicinal and edible cereal crop having various health effects such as strengthening the stomach, promoting digestion and absorption, anti-oxidation, and anti-inflammation (4). In addition, foxtail millet can also be used to treat type II diabetes and cardiovascular diseases (5). Foxtail millet must be dehulled before eating or being used for food processing. However, the storage stability of foxtail millet without a shell is seriously reduced, and it is easy to fade and produces an aging odor, which seriously affects its appearance and nutritional value. Studies have shown that millet aging results from the interaction between the external environment and its components (6). Specifically, the hydrolysis and oxidation of lipids induced by the endogenous lipase and lipoxygenase (LOX) are one of the problems that are prone to occur in the rancidity process of foxtail millet (6, 7). Foxtail millet contains high lipids (> 4%), and the content of polyunsaturated fatty acids accounts for about 85%, which is much higher than that of crops such as corn and rice (8). Lipase could decompose the triglycerides into free fatty acids and reduce the pH value, which causes the rancidity of foxtail millet. LOX oxidizes polyunsaturated fatty acids with a 1-*cis*, 4-*cis*-pentadiene structure, including linoleic and linolenic acid, to form hydroperoxides with the release of highly active oxygen free radicals, thus leading to the outcomes of fading and the generation of aging odor (7). Therefore, exploring effective treatment methods to inactivate lipase and lipoxygenase is of great significance for stabilizing the quality of foxtail millet during storage.

The exploitation of “green,” no additives, and safe, sustainable means, namely, “clean label,” for food processing, has been increasingly researched in recent years. Plasma is mainly composed of positive and negative ions, free radicals, electrons, and the excited state or ground state of atoms (9). Non-thermal plasma technology could be considered a “clean label” owing to no production of residues, which may be an alternative means in the food industry. In recent years, the application of non-thermal plasma in food processing has been widely investigated for the inactivation of endogenous enzymes of food products to extend shelf life and maintain quality (10–14). For example, plasma can inactivate polyphenol oxidase in fruits and vegetables and inhibit the degree of enzymatic browning (15, 16). However, there is a lack of published studies on how this technology affects the foxtail millet and its storage stability.

Shaanxi province is an important foxtail millet production area in China, abounding with famous varieties of Mizhi millet and Ansai millet, cultivated in Yulin and Yan’an city, respectively. In this study, the effects of dielectric barrier discharge-air cold plasma (DBD-ACP) on the lipase, lipoxygenase (LOX) activity, and the appearance, including color, total carotenoids content, morphology, and DPPH free radical scavenging capacity of foxtail millets, i.e., Mizhi and Ansai, were investigated. Additionally, the effects of DBD-ACP on the storage stability of foxtail millets were assessed based on the change of color, total carotenoids, the relative activity of lipoxygenase and lipase, and malondialdehyde (MDA) content during an accelerated storage study for 15 days.

## Materials and methods

### Raw materials

**FIGURE 1:**
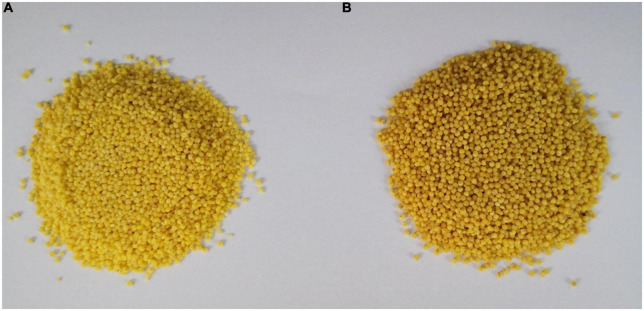
The appearance of foxtail millet. **(A)** Mizhi. **(B)** Ansai.

The current year’s harvested foxtail millets Mizhi (Jingu 21 variety) and Ansai (Yangu 12 variety) (Figure 1) were purchased from a local market in Xi’an, P. R. China. Linoleic acid and *p*-nitrophenyl laurate were from Sigma–Aldrich Co. (St. Louis, MO, USA). 2,2′-diphenyl-2-picrylhydrazyl hydrate (DPPH) and other chemicals used in this study were obtained from Aladdin Chemical Co. (Shanghai, China). All chemicals were of analytical reagent grade. Ultrapure water was used to prepare the samples throughout the whole experiment.

### Dielectric barrier discharge-air cold plasma treatment of foxtail millet

A dielectric barrier discharge plasma system was applied in this study as described by Pan et al. (17), which mainly consisted of two electrodes with an outer diameter of 50 mm (DBD-50), a high-voltage alternating current power source (CTP-2000K), a voltage regulator (Nanjing Suman Electronics Co., Ltd., Nanjing, Jiangsu, China), and two glass dielectric barriers of 1.2 mm in thickness. Foxtail millets with uniform size and color were treated at different voltages (0, 15, 25, and 35 kV) for 0, 2, 4, 8, and 12 min, the frequency was set at 10 kHz, and the discharge spacing was 7 mm. The experimental results were repeated three times, and the average was calculated.

### Instrumental color of foxtail millet

The color parameters lightness (*L*) and yellowness (b*), were determined at six different points of each sample using a digital colorimeter (VS450 Non-contact colorimeter, Xrite Company, USA) after being blank calibration, to reflect the color change of foxtail millet before and after being treated by DBD-ACP.

### Determination of total carotenoid

Total carotenoids of foxtail millet were extracted and analyzed based on a method reported by Abdel-Aal and Rabalski (18) with slight modifications. For the experiment, 0.50 g of foxtail millet flour with different treatments was homogenized in 10.0 mL of water-saturated 1-butanol and kept shaking for 3.0 h at room temperature. The resultants were centrifuged at 6,000 × g for 10 min at 4°C, and the supernatants were collected and filtered through 0.45 μm filters to analyze the total yellow pigment. A UV–Vis spectrophotometer (UV-1800, Shimadzu Scientific Instruments Co., Ltd., Tokyo, Japan) was used to measure the absorbance at 450 nm to measure total carotenoids. All the extraction and analysis of total carotenoids were conducted in dim light to minimize photo-oxidative reactions.

### Determination of lipase and lipoxygenase activity

The extraction and activity determination of lipase were performed according to Lampi et al. (19) with some modifications. For 1.0 h, 1.0 g of ground foxtail millet samples with various treatments were thoroughly mixed with 0.1 M potassium phosphate buffer pH 7.0. After centrifuging the mixture at 8,000 × *g* for 15 min at 4°C, the crude enzyme solution in the supernatants was collected. The activity of lipase and LOX in the supernatant was evaluated using the kits obtained from Nanjing Jiancheng Bioengineering Institute and Suzhou Grace Biotechnology Co., Ltd., China, respectively.

### Diphenyl-2- picrylhydrazyl hydrate free radical scavenging activity

The DPPH radical scavenging activity of foxtail millets was measured by mixing 6 mg of grounded foxtail millets with 2.0 mL of 70% ethanol and 2.0 mL of 0.1 mM DPPH in an ethanolic solution. Afterward, the mixture was shaken and incubated in the dark at ambient temperature for 2.0 h. The absorbance of samples was recorded at 517 nm, and the radical scavenging ability of DBD-ACP treated and untreated millets was expressed as percent free radical scavenging activity.

$$\mathrm{Scavenging}\mathrm{activity}\left(\%\right)=\frac{A_{517\,n\,m}\,b\,l\,a\,n\,k-A_{517\,n\,m}\,s\,a\,m\,p\,l\,e}{A_{517\,n\,m}\,b\,l\,a\,n\,k}$$

### Scanning electron microscopy observation

The surface microstructure of foxtail millets was visualized by scanning electron microscopy (SEM, S-3400N, Hitachi, Japan). The samples of foxtail millets before and after being treated by DBD-ACP at 35 kV for 12 min were fixed on SEM discs with double-sided carbon tape and sputter-coated with platinum for 60 s. The SEM images were magnified 600 times and captured at an accelerating voltage of 10.0 kV.

### Storage analysis for accelerated aging

To evaluate the effects of DBD-ACP under 15 and 25 kV for 4 min on the quality of millets during storage, untreated millets (control) and DBD-ACP treated millets that were kept in Petri dishes in an incubator (40 ± 2°C and 75% RH) for accelerated aging by 15 days. The change of color indexes and the content of total carotenoids, activity of lipoxygenase and lipase, and MDA content were measured every 3 days.

### Malondialdehyde concentration

The MDA concentration of foxtail millet being treated and untreated by DBD-ACP or accelerated storage was evaluated using an MDA determination kit (Beijing Solarbio Science and Technology Co., Ltd.). 0.1 g millet powder was thoroughly mixed with 1.0 mL extracting solution. After incubation in an ice-water bath for 30 min, the mixture was centrifuged at 8,000 × g for 10 min, and the supernatant was kept to determine the MDA concentration following the protocol.

### Statistical analyses

Statistical analysis was performed using OriginPro 8.0 (Origin Lab, Northampton, MA, USA) in triplicate with three independent experiments and results expressed as means ± SD. Analysis of variance (ANOVA) followed by Tukey’s test was carried out using SPSS 22.0 software (IBM, NY, USA), and values were considered significantly different if *p* < 0.05.

## Results and discussion

### Effect of dielectric barrier discharge-air cold plasma on color indexes of foxtail millet

In the CIELAB color representation system, *L* and b* represent the lightness and yellowness of samples, respectively. As shown in Table 1, the *L* and b* values of both Mizhi and Ansai had no significant change (*p* > 0.05) after exposure to DBD-ACP under 15 kV with increasing treatment time. In addition, no significant difference in *L* and b* values were observed for Mizhi and Ansai millet under 25 kV treatment less than 4 min (*p* > 0.05), suggesting that DBD-ACP treatment at 15 kV for 2–12 min or 25 kV for 2–4 min had little effect on the color of millet. However, the levels of *L* value increased after DBD-ACP treatment at high voltage, especially at 35 kV with increasing time (*p* < 0.05), in which the *L* value of Mizhi and Ansai millet increased from 64.23 and 60.51 to 72.5 and 68.68, respectively. The b* value decreased from 49.04 to 46.46 to 42.13 and 40.74, indicating that color changes (bleaching) occurred in millets after being treated with DBD-ACP at 35 kV. Several studies have revealed that DBD-ACP at high voltage induced a significant or slight color change in food, including cherry tomato, Spirulina algae, carrot Juice, Pistachio nuts, banana slices, and grains (14, 20–27). For example, a study conducted by Chaple et al. reported a similar change in color values (*L* and b*) of wheat flour for plasma treated at voltages of 80 kV for 5–30 min (26). Pearl millets were also noticed for the variations in color values produced by atmospheric pressure cold plasma treatment at 40 and 45 kV for 5–15 min (28). These published articles show that DBD-ACP can cause the destruction of pigment materials to some degree. Similarly, the increase in *L* and decrease in b* values in this work are possibly due to the degradation of carotenoids by ozone or free radicals, as carotenoids are responsible for the yellow color of foxtail millet (29).

**TABLE 1:** Effect of DBD-ACP on color indexes of foxtail millets.

Millet	Voltage (kV)	Time (min)	Instrumental color	
			Lightness (*L*)	Yellowness (b*)
Mizhi	0	—	64.23 ± 0.24a	49.04 ± 0.17a
	15	2	64.22 ± 0.27a	49.24 ± 0.19a
		4	64.31 ± 0.31a	49.21 ± 0.31a
		8	64.30 ± 0.26a	49.28 ± 0.25a
		12	64.43 ± 0.19b	49.18 ± 0.28a
	25	2	64.36 ± 0.21a	48.88 ± 0.34a
		4	64.91 ± 0.43ab	48.82 ± 0.32a
		8	65.10 ± 0.31b	48.50 ± 0.31a
		12	67.07 ± 0.25c	46.63 ± 0.24b
	35	2	65.22 ± 0.29b	47.83 ± 0.26b
		4	67.35 ± 0.25c	46.88 ± 0.32c
		8	68.36 ± 0.20d	44.43 ± 0.17d
		12	70.51 ± 0.19e	42.13 ± 0.19e
Ansai	0	—	60.51 ± 0.26a	46.46 ± 0.33a
	15	2	60.61 ± 0.17a	46.48 ± 0.21a
		4	60.69 ± 0.13a	46.35 ± 0.25a
		8	60.70 ± 0.41a	46.39 ± 0.31a
		12	60.68 ± 0.35a	46.39 ± 0.40a
	25	2	60.61 ± 0.31a	46.43 ± 0.14a
		4	60.97 ± 0.33a	46.20 ± 0.16a
		8	62.06 ± 0.26b	45.19 ± 0.26b
		12	63.85 ± 0.23c	43.70 ± 0.24c
	35	2	61.66 ± 0.27b	45.11 ± 0.27b
		4	62.09 ± 0.28b	44.02 ± 0.31c
		8	64.69 ± 0.21c	42.09 ± 0.32d
		12	67.68 ± 0.20d	40.74 ± 0.19e

Different letters within a column are significantly different (*p* < 0.05).

### Effect of dielectric barrier discharge-air cold plasma on total carotenoids in foxtail millet

**FIGURE 2:**
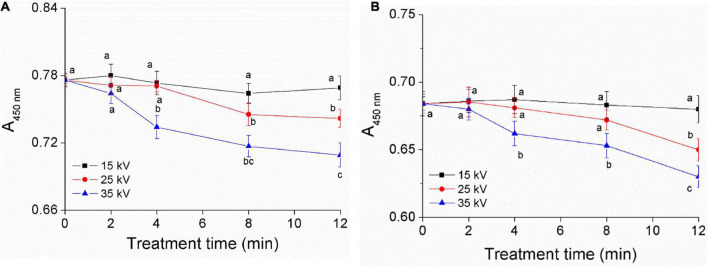
Effect of DBD-ACP treatment on yellow pigment of foxtail millet. **(A)** Mizhi. **(B)** Ansai.

Carotenoids are the main yellow pigment components of foxtail millet, reflecting the quality of millet and having good nutrition function (1, 30). To confirm the effect of DBD-ACP on the pigment of foxtail millet Mizhi and Ansai, total carotenoids were extracted before and after exposure of millets to DBD-ACP under various voltage and treatment times. The absorbance of carotenoids in millet is the highest at 445–450 nm, which could be used as an indicator to reflect its content, qualitatively (30). Figures 2A,B show the changes of *A*_450nm_ of Mizhi and Ansai under different voltages and treatment times, respectively. It can be seen that the *A*_450nm_ of Mizi millet and Ansai millet are 0.78 and 0.68, respectively. Under the voltage of 15 kV, the absorbance of extracted carotenoids in the Mizhi and Ansai millet had no significant change (*p* > 0.05). When the treatment voltage reached 35 kV, the absorbance of yellow pigment was significantly decreased (*p* < 0.05), indicating that carotenoids in millet were damaged to a certain extent. The decrease of carotenoids was also observed by Paixão et al., in which the degradation of carotenoids in siriguela juice was due to exposure to a higher amount of ionized reactive species by plasma (31). Thus, it can be hypothesized that the degradation of superficial carotenoids could be associated with the oxidation of pigments and fading of the color, which is mediated by the presence of oxygen and nitrogen radicals produced by DBD-ACP treatment.

### Effect of dielectric barrier discharge-air cold plasma on enzyme activity in foxtail millet

**FIGURE 3:**
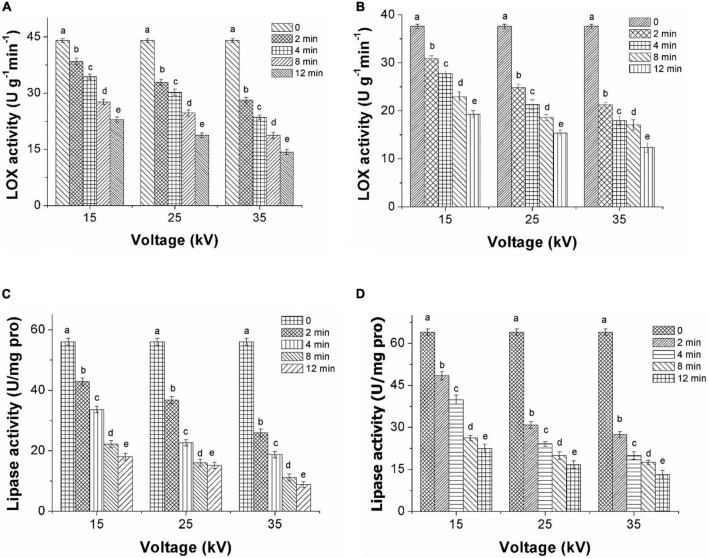
Decrease of LOX and lipase activity of foxtail millet induced by DBD-ACP. **(A,C)** Mizhi. **(B,D)** Ansai.

Inactivation of LOX treated by DBD-ACP under voltages of 15, 25, and 35 kV with various treatment times was initially evaluated. As shown in Figure 3A, the activity of LOX in foxtail millet Mizhi gradually decreased after exposure to DBD-ACP treatment (*p* < 0.05). For example, when DBD-ACP treated millets at 15 kV for 2–12 min, the relative activity of LOX was decreased from 44.0 U g^–1^ min^–1^ to 38.4, 34.3, 27.6, and 22.9 U g^–1^ min^–1^. For DBD-ACP treatment at 25 kV, the relative activity of LOX was 32.8 U g^–1^min^–1^ at 2 min, and the figure decreased sharply to 30.1, 24.6, and 18.7 U g^–1^ min^–1^ after 4, 8, and 12 min, respectively. Correspondingly, lower activity of LOX, i.e., 28.1, 23.5, 18.7, and 14.3 U g^–1^min^–1^ were obtained for foxtail millet Mizhi treated by DBD-ACP treatment at 35 kV for 2, 4, 8, and 12 min, respectively. The relative activity of LOX in the Ansai millet is shown in Figure 3B, exhibiting a similar behavior with the increase of voltage and time, indicating that DBD-ACP has a significant inactivation effect on LOX in millets. Since LOX is one of the key enzymes that promote the aging of millet (8), the inactivation of LOX by DBD-ACP may play a positive role in delaying the aging process of millet.

Figures 3C,D show the inactivation of lipase in the foxtail millet of Mizhi and Ansai, respectively. The lipase activity of millets after being treated by DBD-ACP decreased rapidly with the increase in voltage and treatment time (*p* < 0.05). For example, the lipase activity in the Mizhi millet was inactivated from 56.0 to 18.0 U/(mg pro), 15.1 and 8.8 U/(mg pro), decreased by DBD-ACP treatment at 35 kV for 2–12 min to 67.8, 73.0, and 84.3% in comparison with the untreated millet. These results were in accordance with several previous studies that DBD-ACP treatment is effective in enzymes, including proteinase, pectinase, and alkaline protease (11, 32, 33). The enzymatic inactivation mechanism of DBD-ACP is considered to cause conformational changes, including lower the proportion of α-helix structure due to reacting with a variety of hydrogen peroxide (H_2_ O_2_), ozone (O_3_) and nitrate ions (NO_3_^–^) as well as hydroxyl radical (OH^–^), superoxide (O_2_^–^), and singlet oxygen generated by DBD-ACP with the side chain of the enzyme molecules (34–36).

### Effect of dielectric barrier discharge-air cold plasma on antioxidant activity of foxtail millet

**FIGURE 4:**
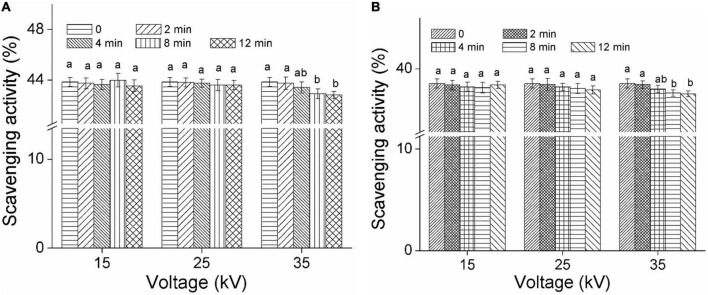
Effect of DBD-ACP on DPPH free radical scavenging activity (%) of foxtail millet. **(A)** Mizhi. **(B)** Ansai.

Figures 4A,B show the DPPH radical scavenging ability of foxtail millet Mizhi and Ansai by DBD-ACP treatment, respectively. As can be seen from the figures, the DPPH radical scavenging ability of Mizhi and Ansai did change insignificantly under plasma treatment (15 and 25 kV) (*p* > 0.05), and maintained about 43.8 and 38.5%, respectively. This result implied that DBD-ACP treatment at relatively low voltage did not change the antioxidant activity. The results of this study were consistent with a work conducted by Tolouie et al. (16), who reported the DPPH free radical scavenging activity of wheat germ did not alter significantly during DBD-ACP treatment. Similar results were reported by Amini and Ghoranneviss (37) and Ramazzina et al. (14), who found that DBD-ACP treatment did not affect the antioxidant capacity of fresh walnuts and kiwifruit. However, the scavenging ability of DPPH free radical of millets after exposure to DBD-ACP at 35 kV showed a slight decrease, significantly (*p* < 0.05) when DBD-ACP treatment for 8 and 12 min was compared to the control. These findings agree with grape juice and chokeberries that, when treated with cold plasma show a lower DPPH radical scavenging ability at higher treatment voltage or time (38, 39). Therefore, it can be inferred that the effect of DBD-ACP on DPPH radical scavenging ability or antioxidant activity is probably dependent on the plasma processing parameters.

### Morphological observation of foxtail millet

**FIGURE 5:**
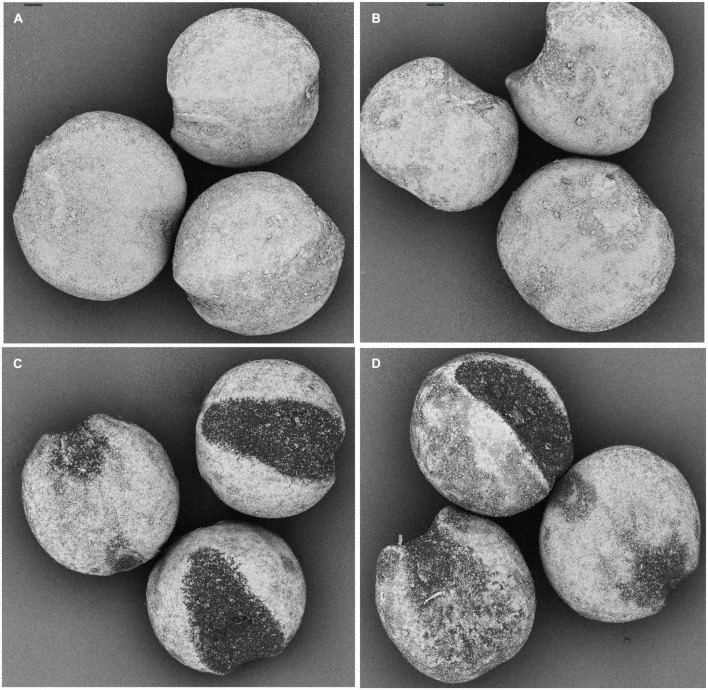
Morphological images of untreated foxtail millet, **(A)** Mizhi and **(C)** Ansai, and after being treated by DBD-ACP at 35 kV for 12 min, **(B)** Mizhi, **(D)** Ansai.

The surface morphology of foxtail millets before and after being treated by DBD-ACP at 35 kV for 12 min was observed by SEM. Figures 5A,C show the surface of the untreated Mizhi and Ansai millet, describing the intact morphology of dehulled millet with evident furrows caused by the shelling treatment. The DBD-ACP treated millets show a morphology with a well-defined texture and large grooves with no significant cracks were observed (Figures 5B,D). These results were consistent with a work done by Thirumdas et al. (40), reporting that brown rice showed fissures and hollow depressions under cold plasma treatment. This may be due to differences in the texture between the rice and foxtail millet. Therefore, it could be concluded that DBD-ACP treatment does not produce mechanical damage to the surface of foxtail millets under these conditions.

### Color changes of foxtail millet during storage

**FIGURE 6:**
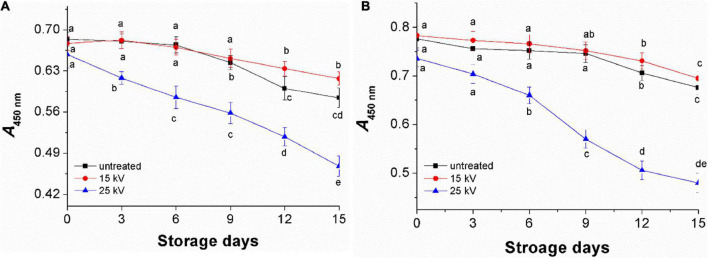
The changes of yellow pigment of foxtail millets during accelerated agingstorage. **(A)** Mizhi. **(B)** Ansai.

Untreated foxtail millets (control) and treated at 15 and 25 kV for 4 min were kept for accelerated aging storage by 15 days. The results showed a moderate increase in *L* and a decrease in b* values of untreated millets (Table 2). Compared to the control, millets treated at 15 kV had better color indexes, indicating that DBD-ACP treatment at 15 kV has a positive effect on millets on keeping a better appearance. However, much more significant variations were observed in the millets treated by DBD-ACP at 25 kV. Similar results were found in the changes of *A*_450__*nm*_ representing the total carotenoids, where millets exposed to 25 kV showed a significant reduction during storage compared to the control and millets treated at 15 kV (Figures 6A,B). These results may be related to ozone (O_3_), nitrate ion (NO_3_^–^), superoxide (O_2_^–^), and singlet oxygen produced by DBD-ACP at high voltage remaining on the surface of millet, which accelerates the destruction of the millet pigment in the aging storage (14).

**TABLE 2:** Changes in color indexes of foxtail millets with different treatment.

	Lightness (*L*)			Yellowness (b*)		
	**Mizhi millet**					
Storage days	0	15	25	0	15	25
0	64.33 ± 0.21a	64.41 ± 0.30a	64.94 ± 0.18a	49.19 ± 0.21a	49.30 ± 0.22a	48.45 ± 0.24a
3	64.54 ± 0.25ab	64.42 ± 0.41a	65.41 ± 0.26a	48.94 ± 0.26a	49.42 ± 0.23a	47.03 ± 0.40b
6	64.91 ± 0.42b	64.50 ± 0.22a	65.93 ± 0.27a	48.04 ± 0.30b	49.32 ± 0.19a	46.72 ± 0.32b
9	65.22 ± 0.22bc	64.53 ± 0.26a	66.48 ± 0.27b	47.47 ± 0.25b	49.06 ± 0.35a	46.0 ± 0.27a
12	65.58 ± 0.28c	65.18 ± 0.24b	67.63 ± 0.42c	46.89 ± 0.21c	48.71 ± 0.23b	44.58 ± 0.34d
15	66.35 ± 0.19d	65.77 ± 0.31c	69.80 ± 0.30d	46.07 ± 0.22d	47.01 ± 0.31c	42.03 ± 0.25e
	**Ansai millet**					
0	60.56 ± 0.15a	60.62 ± 0.22a	62.92 ± 0.26a	46.52 ± 0.23a	46.43 ± 0.21a	45.22 ± 0.24a
3	60.47 ± 0.11a	60.72 ± 0.29a	63.83 ± 0.32b	46.60 ± 0.31a	46.39 ± 0.40a	45.01 ± 0.26a
6	61.05 ± 0.13b	60.62 ± 0.42a	64.75 ± 0.31c	46.01 ± 0.11b	45.75 ± 0.31b	44.41 ± 0.32b
9	62.17 ± 0.25c	61.13 ± 0.35a	65.39 ± 0.32d	45.44 ± 0.20c	45.01 ± 0.26c	42.63 ± 0.11c
12	63.18 ± 0.36d	62.16 ± 0.19b	66.88 ± 0.34e	44.58 ± 0.22d	44.85 ± 0.34c	41.46 ± 0.24d
15	63.37 ± 0.26d	62.73 ± 0.22c	68.15 ± 0.40f	43.78 ± 0.18e	44.23 ± 0.27d	39.44 ± 0.26e

Different letters within a column are significantly different (*p* < 0.05).

### Activity of lipoxygenase and lipase during storage

**FIGURE 7:**
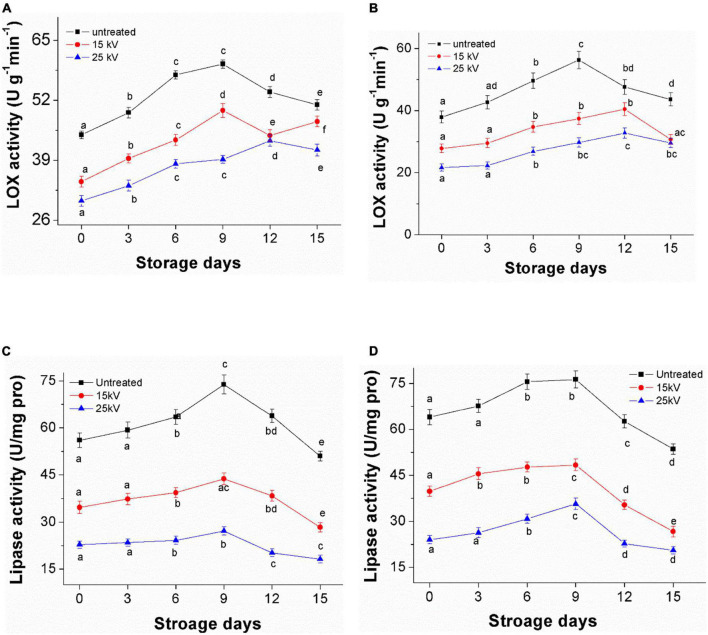
Changes of LOX and lipase activity of foxtail millet during accelerated aging storage. **(A,C)** Mizhi. **(B,D)** Ansai.

Figure 7 presents the LOX and lipase activities of millets during 15 days of accelerated aging storage. LOX and lipase activity were observed to increase gradually in the first 9 days of storage and then to decrease regardless of treatments. For example, the activity of LOX for untreated Mizhi and Ansai millet during storage was 44.5 and 37.9 U g^–1^ min^–1^ on day zero, increasing to a minimum of 59.8 and 56.3 U g^–1^ min^–1^ after 9 days of storage, respectively (Figures 7A,B). The lipase activity for untreated Mizhi and Ansai millet during storage was only 56.0 and 64.0 U/(mg pro), increasing to a minimum of 73.8 and 76.3 U/(mg pro) after 9 days of storage, respectively (Figures 7C,D). However, the treated millets by DBD-ACP at 15 and 25 kV were found to have maintained a significantly lower value of LOX and lipase activity than that of the control. These results concur with a previous study by Tolouie et al. (16), in which DBD-ACP-treated wheat germ was found to maintain a low LOX and lipase activity during storage.

### Production of malondialdehyde

**FIGURE 8:**
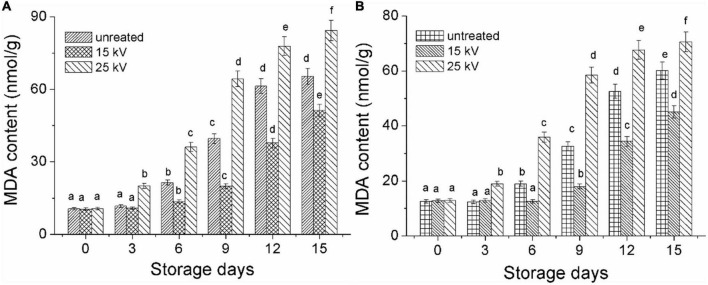
Changes in MDA content of foxtail millet during accelerated aging storage. **(A)** Mizhi. **(B)** Ansai. Different letters with the same column are significantly different (*p* < 0.05).

As shown in Figure 8A, the MDA content of Mizhi millet that was untreated by DBD-ACP was found to be slowly increased, from 12.5 to 18.9 mg/g, 32.5 and 60.1 mg/g on the days of 0, 6, 9, and 15 (*p*< 0.05). Whereas millet treated with DBD-ACP at 15 kV had significantly lower MDA content than the control. The accumulation of MDA is related to the oxidation of lipids, especially unsaturated fatty acids induced by LOX and lipase. Consequently, the MDA contents of the 15 kV treated millet were low during the accelerated storage (41). However, it was found that MDA content drastically increased for millets treated by DBD-ACP at 25 kV. This increase in MDA content be associated with the reactive species and hydroxyl free radicals generated by DBD-ACP at high voltage that initiate lipid oxidation by attacking various compounds, including unsaturated fatty acids. These results concur with previous reports in which cold plasma increased the MDA content of pistachio walnuts and peanuts at different powers (24, 40). Ansai millets with different treatments also exhibited similar results during the same storage conditions (Figure 8B). These results, along with the changes in color indexes, total carotenoids, and antioxidant activity indicated that nutritional losses of foxtail millets were associated with ROS-triggered lipid peroxidation and destruction of millet pigment, which was greatly alleviated by DBD-ACP treatment under severe conditions, suggesting that when using DBD-ACP to delay millet aging, appropriate treatment conditions are required.

## Conclusion

This work investigated the effects of DBD-ACP on the quality and storage stability of foxtail millets. DBD-ACP treatment was found to cause a noticeable decrease in the activity of lipoxygenase and lipase of foxtail millets. For example, LOX and lipase activity decreased from 44.0 to U g^–1^min^–1^, 56.0–8.8 U/(mg pro) by DBD-ACP treatment at 35 kV for 12 min, respectively. Moreover, DBD-ACP has no significant impact on foxtail’s antioxidant activity and surface morphology. However, it has a certain destructive effect on the total carotenoids that affect the color of foxtail millet if the voltage is at 35 kV for 4–12 min or 25 kV for 8–12 min. After being treated by DBD-ACP under 15 and 25 kV for 4 min, the stability of foxtail millets was determined during accelerated storage analysis for 15 days. Compared to the controls, millets treated by DBD-ACP at 15 and 25 kV were found to have maintained a significantly lower value of LOX and lipase activity during the accelerating aging storage. Additionally, millets treated by DBD-ACP at 15 kV exhibited a better color index, higher total carotenoids, and lower MDA content during storage. Comparatively, DBD-ACP at 25 kV induced damages in color, total carotenoids, and higher MDA content of millets suggesting side effects occurred due to reactive oxygen species. The elucidation of DBD-ACP under suitable treatment conditions could be applied to stabilize the quality of the foxtail millet. These results implied that DBD-ACP is an underlying approach for the storage of foxtail millets. Further work should be done to enhance the understanding of DBD-ACP on foxtail millets and to evaluate the feasibility of using DBD-ACP to delay the shelf life of dehulled foxtail millets.

## Data availability statement

The original contributions presented in this study are included in the article/supplementary material, further inquiries can be directed to the corresponding authors.

## Author contributions

L-HW: conceptualization, methodology, formal analysis, data curation, and writing—original draft. ZL: methodology, formal analysis, and data curation. JQ: methodology, formal analysis, and visualization. YH: resources, methodology, and supervision. X-AZ: resources, visualization, supervision, project administration, and funding acquisition. RMA: writing—review and editing. All authors contributed to the article and approved the submitted version.
